# Barriers to Institutional Delivery in Urban Poor Society: Findings From Indonesia’s National Survey

**DOI:** 10.34172/jrhs.9131

**Published:** 2025-09-15

**Authors:** Marizka Khairunnisa, Agung Dwi Laksono, Leny Latifah, Mohamad Samsudin, Taufiq Hidayat, Diah Yunitawati

**Affiliations:** ^1^Research Center for Public Health and Nutrition, Research Organization for Health, National Research and Innovation Agency, Bogor, Indonesia

**Keywords:** Institutional delivery, Institutional birth, Maternal health, Urban poor, Public health

## Abstract

**Background:**

The urban poor represent a vulnerable population within society, particularly in terms of maternal health. Economic and access-related limitations often prevent this group from accessing healthcare services, especially in the institutional delivery process. This study aimed to analyze the barriers to institutional delivery among Indonesia’s poor urban society.

**Study Design:**

This study employed a cross-sectional design.

**Methods:**

Data were obtained from the 2023 Indonesian Health Survey, including 7,548 participants. Eight independent variables were analyzed, including age, education, marital status, employment, wealth, insurance, and parity, with institutional delivery used as the dependent variable. Binary logistic regression was employed for analysis.

**Results:**

Approximately 38.1% of Indonesian pregnant women had non-institutional deliveries. All age groups showed a higher likelihood of non-institutional delivery compared to those aged≥45. Lower education levels were associated with a heightened probability of choosing non-institutional delivery. Married women were 0.704 times less likely than divorced or widowed women to give birth in non-institutional settings (AOR: 0.704; 95% CI: 0.693-0.716). Unemployed women had 1.218 times higher likelihood of engaging in non-institutional delivery compared to employed women (AOR: 1.218; 95% CI: 1.1210-1.226). The poorest women were 0.973 times less likely than the poorer group to have non-institutional delivery (AOR: 0.973; 95% CI: 0.967-0.980). Uninsured women were 2.364 times more likely than insured women to give birth outside of healthcare institutions(AOR: 2.364; 95% CI: 2.345-2.379). Women with all other parity levels were less likely than grand multiparous women to have non-institutional deliveries.

**Conclusion:**

Seven barrier factors to institutional delivery were younger age, low education, divorced/widowed marital status, unemployment, lower wealth status, lack of insurance, and grand multiparity.

## Background

 Maternal mortality rate (MMR) refers to the number of women who die from pregnancy-related causes or postpartum problems per 100,000 live births.^1^ Between 2000 and 2020, the global MMR declined by 34%, from 342 to 223 deaths per 100,000 live births.^2^ Nonetheless, low- and lower-middle-income nations accounted for 95% of maternal mortality in 2020. During the same period, the MMR significantly decreased in Eastern Europe (from 38 to 11) and South Asia (from 408 to 134), while Sub-Saharan Africa recorded a 33% reduction.^2^ Despite these improvements, Indonesia’s MMR remained high at 189 per 100,000 live births in 2020, far above the Sustainable Development Goals (SDGs) target of 70 per 100,000 live births by 2030. Although Indonesia’s MMR dropped by 45% between 2010 and 2015,^3^ the rate remains high compared to upper-middle-income countries, other Southeast Asian countries, and World Health Organization (WHO) benchmarks.^4^

 Another critical indicator of a successful health program is the infant mortality rate (IMR). Globally, IMR declined from 65 to 29 deaths per 1,000 live births between 1990 and 2018, with the highest rates reported in Africa.^5^ In Indonesia, IMR fell from 47 per 1,000 live births in 2000 to 16.85 in 2020, primarily due to improved maternal healthcare services.^6^

 To lower MMR and IMR, the Indonesian Ministry of Health issued Regulation Number 97 of 2014, mandating childbirth in health facilities.^7^ However, challenges persist in ensuring the availability of trained medical personnel, due to social, cultural, and economic barriers that hinder access to healthcare for pregnant women and infants.^8^ Although maternal deaths are largely preventable, they remain concentrated in poorer countries.^9^ Limited access to reproductive healthcare caused by poverty, geographic barriers, insufficient education, and cultural influences exacerbates the issue.^10^ Research indicates that in over 50% of countries, the prevalence of facility-based delivery remains below 70%,^11^ including Indonesia, with a prevalence of 55.6%.^12^ Factors such as socio-demographic characteristics, educational level, health service-related issues,^13^ and routine healthcare appointments^14^ affect the decision to give birth at a healthcare facility.

 Globally, urbanization is expected to persist, with nearly two-thirds of the world’s population predicted to live in urban regions by the mid-21st century.^15^ This leads to several health challenges, including maternal and child health.^16^ Within urban areas, the poor are more vulnerable to these health problems than the rich. Economic limitations make it more difficult for low-income groups to access nutritious food and force them to live in vulnerable areas full of pollution, densely populated, and lacking clean water sources.^17^ Individuals living in poverty who get ill and incur expenses for treatment and medical care are at heightened risk of further impoverishment. It explains the reasons behind the limited access of the impoverished to healthcare facilities or their reluctance to utilize them,^18^ including limited access to institutional delivery services during childbirth. Based on this rationale, the present study aimed to analyze the barriers to institutional delivery among Indonesia’s urban poor population.

## Methods

###  Data source and study design

 This study utilized secondary data from the 2023 Indonesian Health Survey conducted by the Ministry of Health of the Republic of Indonesia. The survey employed a multistage stratified sampling design to produce representative estimates at national and regional levels. In the first stage, census blocks (CBs) were randomly selected using the probability proportional to size method. A total of 34,500 CBs were randomly chosen from the national master sampling frame provided by Statistics Indonesia (BPS), serving as primary sampling units, and stratified by urban-rural classification and districts to ensure national representativeness.

 In the second stage, a fixed number of households within the selected CB were randomly selected using systematic random sampling from the available household list. In the third stage, all eligible individuals within the selected households were interviewed using a standardized survey questionnaire.^19^ At this stage, sample selection has gone through the randomization step. This design and these stages were designed to reduce selection bias and ensure data representativeness at the national and sub-national levels.

 This study used secondary data, which involved approximately 690,000 households as the initial target population, covering regular households and households with toddlers. A regular household refers to a residential unit consisting of one or more individuals living together in one dwelling and typically sharing meals prepared in a single kitchen. Households with toddlers are defined as regular households with at least one child aged 0–59 months (toddler) at the time of the survey. For this study, individual-level data were extracted from regular households. Individual respondents were chosen based on specific criteria relevant to the research objectives. For this analysis, a sample of 7,548 individuals was identified through screening based on research criteria, which included only poor urban Indonesian women who had given birth in the past five years. The 2023 Indonesian Health Survey provided sampling weights, which were used in all analyses to ensure the representativeness of the estimates for the target population. The analysis was limited to poor urban areas, as defined by Statistics Indonesia. The study population was selected based on area codes combined with poverty data, specifically covering the poor urban population.

 Data were collected between May and July 2023 through face-to-face interviews using structured questionnaires administered by trained enumerators. The use of computer-assisted personal interviewing, field supervision, and quality control measures helped minimize both interviewer and recall bias. The participation rate obtained was 91.49%.

###  Setting

 This study was conducted in urban areas with populations classified as economically disadvantaged, following the urban-rural classifications established by Statistics Indonesia. The research assessed a family’s wealth status by examining the fifth most valuable item in the household.

 The household wealth index was calculated using the principal component analysis (PCA) method, based on a range of asset indicators such as ownership of durable goods, housing characteristics, and sanitation facilities. The data were standardized prior to PCA, with the first principal component used to calculate the index score. This score was then used to group households into socioeconomic quintiles. The resulting index demonstrated good construct validity, as it could theoretically differentiate household welfare levels and demonstrated adequate reliability, as indicated by eigenvalues, factor loadings, and sampling adequacy measures such as Kaiser–Meyer–Olkin (KMO).^19^

 The questions used to measure family wealth in this survey focused on the ownership of durable goods, such as houses, cars, motorcycles, and refrigerators, as well as the main types of building materials used in different parts of the house. The wealth index was normalized at the national level to offer a more comprehensive measure of economic status by incorporating household assets and their conditions. This wealth index provides a more stable and reliable measure of economic status than income or expenditure, which can be unstable and difficult to measure accurately, particularly in low-income or informal settings.^20^ The variables related to economic status were processed through factor analysis, resulting in a correlation matrix. The number of factors retained was determined based on eigenvalues using the PCA method. Only variables with correlation values above 0.3 were used to predict economic status.

 The ownership variables analyzed by PCA included home ownership status, type and power of lighting in the house, and ownership of durable goods such as gas cylinder ( ≥ 5.5 kg), washing machine, refrigerator, mobile phone, air conditioner, water heater, computer/laptop, flat screen TV ( ≥ 30 inches), private vehicle, and gold/jewelry ( ≥ 10 g). Other variables included livestock ownership, primary cooking fuel, access to defecation facilities, and the main type of construction materials used for the roof, ceiling, floor, and walls. PCA unified these variables into a single measure, referred to as the ownership index.^21^

 Factor analysis was applied to develop this ownership index, serving as a proxy for household economic status. The model’s validity was assessed using Bartlett’s Test of Sphericity and the KMO measure. Only variables with inter-variable correlations > 0.3, KMO > 0.5, and significance levels < 0.05 were retained for analysis. Variables with low correlations were gradually eliminated until only those meeting the criteria remained. Subsequently, PCA was conducted, and components with eigenvalues ≥ 1 were selected for further analysis. The results showed that the combination of variables could explain more than half of the variance in household economic status. Final index scores were then used to group all households into five socioeconomic quintiles: poorest, poorer, middle, richer, and richest.^19^ For this study, households classified as poorest and poorer were grouped together under the term “poor”.

###  Dependent variable

 The dependent variable in this study was institutional delivery, defined as childbirth occurring in healthcare facilities such as hospitals or health centers. Institutional delivery was split into “yes” and “no.”

###  Independent variables

 The research considered seven factors: age group, marital status, education level, employment status, wealth, health insurance ownership, and parity (number of previous births). Age groups were categorized as: ≤ 19, 20–24, 25–29, 30-34, 35–39, 40–45, and ≥ 45. Marital status was classified as either married or divorced/widowed. Educational attainment was divided into five levels: no formal education, elementary school, junior high school, senior high school, and college.

 Employment status was classified as either employed or unemployed. Wealth status focused on the poorest socioeconomic group. Health insurance was divided into two groups: those with and without insurance. Parity is divided into three types: primiparous ( ≤ 1 birth), multiparous (2-4 births), and grand multiparous ( > 4 births).

###  Data analysis 

 A bivariate analysis using the chi-square test was conducted to assess the relationship between each independent variable and the dependent variable. Subsequently, a binary logistic regression model was applied, with results reported as adjusted odds ratios (AORs) and 95% confidence intervals (CIs). All data analyses were performed using IBM SPSS version 26. ArcGIS 10.3 (ESRI Inc., Redlands, CA, USA) was used to map non-institutional delivery rates among urban poor populations, using administrative boundaries defined by Statistics Indonesia.

## Results

**Figure 1:**
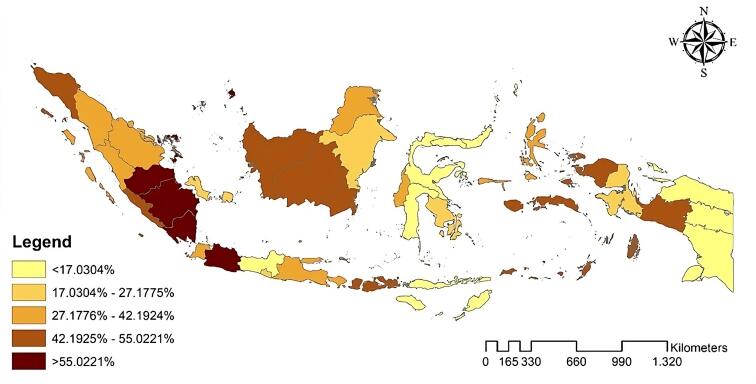
Geographic depiction of non-institutional childbirth among the urban poor in selected provinces of Indonesia, 2023. *Source.* Visualization by the Authors

 According to the data, 38.1% of pregnant women in Indonesia delivered their babies outside formal healthcare facilities. Figure 1 displays the geographic distribution of non-institutional deliveries among poor urban populations across several Indonesian provinces in 2023, showing no spatial pattern by province.

Table 1 presents the bivariate analysis results. The age group of 19 or younger had the highest proportion of non-institutional deliveries. According to marital status, the ratio of divorced/widowed women is higher than that of married women in the non-institutional childbirth group. Meanwhile, according to educational attainment, women with no formal education accounted for the highest proportion within the non-institutional delivery group. Furthermore, unemployed women exhibited a higher percentage of non-institutional deliveries than employed women. Non-institutional deliveries were more prevalent among women in the poorest wealth category than those in the poorer category, while uninsured women had higher institutional delivery rates than insured women. Among parity categories, multiparous women had the highest proportion of non-institutional deliveries.

**Table 1:** Descriptive statistics of participants’ characteristics (N = 7,548)

**Demographic characteristics**	**Institutional delivery**		***P*** ** value**
	**Yes, (n=4,853)**	**No, (n=2,695)**	
Age group (year)			0.001
≤ 19	56.5%	43.5%	
20-24	59.1%	40.9%	
25-29	61.7%	38.3%	
30-34	61.6%	38.4%	
35-39	63.4%	36.6%	
40-44	64.9%	35.1%	
≥ 45	69.7%	30.3%	
Marital status			0.001
Married	62.1%	37.9%	
Divorced/Widowed	55.6%	44.4%	
Education level			0.001
No formal education	54.2%	45.8%	
Primary school	54.5%	45.5%	
Junior high school	59.5%	40.5%	
Senior high school	69.5%	30.5%	
College	74.7%	25.3%	
Employment status			0.001
Unemployed	60.0%	40.0%	
Employed	66.9%	33.1%	
Wealth status			0.001
Poorest	60.7%	39.3%	
Poorer	62.4%	37.6%	
Health insurance			0.001
Uninsured	46.1%	53.9%	
Insured	67.5%	32.5%	
Parity			0.001
Primiparous	64.6%	35.4%	
Multiparous	60.6%	39.4%	
Grand multiparous	62.4%	37.6%	

 A collinearity test was conducted in the second part of the study, which showed minimal correlations among independent variables, with each variable meeting the acceptable threshold correlation of ≥ 0.10. All variance inflation factor values were below 10.00, confirming the absence of multicollinearity in the regression model.

Table 2 presents the binary logistic regression analysis results, using non-institutional delivery as the reference in the final stage. Logistic regression analysis was performed to identify factors independently associated with the outcome. All variables with a *P*< 0.25 in the bivariate analysis were included in the multivariate model. The OR of non-institutional delivery variables in Table 2 was obtained by including all independent variables in the analysis, including age, education, marital status, employment, wealth, insurance coverage, and parity. The findings indicated that all age groups had a higher likelihood of non-institutional delivery compared to those aged 45 and above. Furthermore, an inverse relationship was observed between education level and the likelihood of non-institutional delivery. Married women were 0.704 times less likely to deliver in non-institutional settings compared to divorced or widowed women (AOR: 0.704; 95% CI: 0.693-0.716). Additionally, unemployed women had a 1.218 times higher likelihood of non-institutional delivery than employed women (AOR: 1.218; 95% CI: 1.1210-1.226). Regarding socioeconomic status, women in the lowest wealth group had 0.973 times lower odds of non-institutional delivery compared to those in the poorer group (AOR: 0.973; 95% CI: 0.967-0.980). In terms of insurance coverage, uninsured women had 2.364 times higher odds of giving birth outside institutional settings compared to insured women (AOR: 2.364; 95% CI: 2.345-2.379). Moreover, all parity types were less likely to deliver in non-institutional settings compared to grand multiparous women.

**Table 2:** Logistics regression of institutional delivery in urban poor society (N = 7,548)

**Variables**	**Adjusted OR (95% CI)**	***P*** ** value**
Age group (year)		
≤ 19	1.994 (1.933, 2.057)	0.001
20-24	2.011 (1.964, 2.059)	0.001
25-29	1.735 (1.696, 1.775)	0.001
30-34	1.594 (1.558, 1.630)	0.001
35-39	1.353 (1.323, 1.385)	0.001
40-44	1.274 (1.243, 1.305)	0.001
≥ 45	Ref.	
Education		
No education	2.149 (2.094, 2.206)	0.001
Primary school	2.089 (2.053, 2.125)	0.001
Junior high school	1.676 (1.647, 1.705)	0.001
Senior high school	1.153 (1.133, 1.172)	0.001
College	Ref.	
Marital status		
Married	0.704 (0.693, 0.716)	0.001
Divorced/Widowed	Ref.	
Employment		
Unemployed	1.218 (1.210, 1.226)	0.001
Employed	Ref.	
Wealth		
Poorest	0.973 (0.967, 0.980)	0.001
Poorer	Ref.	
Insurance		
Uninsured	2.364 (2.349, 2.379)	0.001
Insured	Ref.	
Parity		
Primiparous	0.714 (0.702, 0.726)	0.001
Multiparous	0.924 (0.910, 0.939)	0.001
Grand multiparous	Ref.	

## Discussion

 The result indicated that women in all age groups were more likely to have non-institutional deliveries compared to those aged 45 and above. Younger mothers may face challenges such as a lack of experience or support, and they may also be less inclined to seek institutional care due to socioeconomic constraints or a lack of awareness.^22^ Mothers who are unaware or unprepared for labor complications are less likely to choose delivery in healthcare facilities.^23^ A study in Nigeria showed that younger women utilized health facilities less frequently than older women.^24^ Young women, especially first-time pregnant mothers, may have a limited understanding of the benefits of using healthcare facilities and the risks associated with home births. They might be more stressed and less prepared for their pregnancies, leading to their preference for home delivery. Conversely, the probability of institutional childbirth increases with maternal age.^25^ It is probably due to the greater risks of pregnancy complications. Older maternal age adversely affects pregnancy parameters, such as increased gestational diabetes, gestational hypertension, preeclampsia, premature birth, and cesarean section.^26^

 A study by Oumer in the Delgi District, Northwest Ethiopia, found contrasting results, reporting that young mothers (aged 23-27) more frequently utilized institutional childbirth services.^27^ In Ethiopia, the proportion of young women aged 15 to 24 years who had an institutional delivery increased from 6% in 2000 to 40.1% in 2016.^28^ The factors contributing to this trend include improved economic status, education, frequency of antenatal care visits, and urban residency. Similarly, Ugandan women aged 15–19 were reported to give birth in hospitals at twice the rate of those aged 40–49, underscoring the influence of other factors such as education, economic status, and geographical accessibility.^29^

 A lower level of education was strongly correlated with an increased probability of non-institutional delivery. Limited access to health-related information and healthcare services in general, along with potential financial constraints, is often correlated with lower educational attainment.^30^ Research by Bolarinwa supports this finding, indicating that women without formal education and those from lower economic status utilize healthcare facilities less frequently, preferring home delivery instead.^24^ Young women, especially those without formal education, may have limited awareness of the benefits of healthcare facilities for childbirth, and those from underprivileged backgrounds may struggle to afford the associated costs.^31^ Conversely, mothers with higher levels of education are more likely to give birth in healthcare facilities, as they tend to have a better understanding of reproductive health and the importance of utilizing such services.^25^ Education can raise awareness of the benefits of professional medical care and the risks associated with home childbirth. Therefore, improving women’s education should be part of strategies aimed at enhancing childbirth in health institutions.

 This study also revealed that married women were more inclined to opt for institutional delivery than divorced or widowed women. Research among women in Northwest Ethiopia found similar results, showing that divorced women had a lower likelihood of institutional delivery than married women.^27^ Married women are more likely to choose institutional delivery due to better access to social support, financial resources, and transportation, which collectively facilitate the use of health services during childbirth. Marital status is frequently associated with socioeconomic variables, and married women may have more stable socioeconomic circumstances that allow them to access healthcare facilities.^27^

 In the current study, the sample exclusively consisted of married or divorced/widowed women. However, a study in the Philippines reported a significant proportion of never-married women and revealed that these women had higher odds of using institutional delivery than both married or divorced/widowed women.^32^ The greater likelihood of never-married women choosing institutional delivery may reflect higher levels of empowerment and autonomy in making health-related decisions.

 Moreover, according to employment status, unemployed women were more likely to have non-institutional delivery than their employed counterparts. Employment status often correlates with higher income level and better access to healthcare services. Unemployed women may face financial constraints or lack health insurance, which limits their ability to afford institutional delivery.^33^ Previous research in the Gambia also found a similar result, where employed women utilized institutional delivery services more frequently than unemployed women.^34^

 The result revealed that the poorest women engaged in non-institutional deliveries at a lower rate than the poorer women. This finding contrasts with the results from previous research, which found that economic status is a significant barrier to accessing institutional care.^8^ In urban contexts, this result may suggest that even the poorest women could have relatively better physical access to healthcare facilities due to their proximity and may benefit more from targeted urban health interventions or subsidized services. These women in urban settings often become the primary focus of government-supported maternal health programs. As a result, despite their economic limitations, they may have greater access and exposure to institutional health services through targeted outreach, subsidies, and health promotion activities led by midwives and community health workers.^35^ Conversely, women in the poorer group might fall into the coverage gap. They may not qualify for target recipients of government assistance but still face significant financial burdens when seeking private care or even public services that impose out-of-pocket fees for non-subsidized patients or fail to utilize institutional services due to additional expenses involved in the process.^36^ These findings underscore the importance of understanding how urban poverty intersects with healthcare access and how policy targeting may unequally impact different subgroups within the poor population.

 Meanwhile, regarding health insurance status, uninsured women had a higher probability of undergoing non-institutional childbirth compared to insured women. Without health insurance, the cost of institutional delivery can be prohibitively expensive, discouraging the use of such services.^33^ Similarly, Rodrigo-Gallardo et al^37^ reported that women with health insurance demonstrated a greater propensity for utilizing institutional delivery services, including prenatal and maternal care. A study in Indonesia also found that the majority of uninsured pregnant women preferred home births.^38^ Health insurance plays a critical role in reducing financial barriers to accessing maternal health services. The cost of institutional delivery can be high and would become unaffordable without health insurance.^37^ Consequently, those who cannot afford healthcare services are more likely to opt for non-institutional childbirth. Therefore, health insurance can enhance both the utilization and provision of maternal health services, particularly in increasing the number of maternal deliveries at health facilities.

 Furthermore, the study found that all parity levels were less likely than grand multiparous women to deliver in non-institutional settings. Women with many previous pregnancies might face more complex medical histories or increased socioeconomic challenges that influence their delivery place decisions. Rodrigo-Gallardo et al^37^ also noted that women with three or more previous deliveries were less likely to utilize institutional childbirth services compared to those delivering their first or second child. Previous delivery experiences may have enhanced the grand multiparous mother’s confidence to give birth at non-institutional facilities.^39^ In contrast, primiparous women often prefer delivery in medical facilities monitored by qualified healthcare professionals for emergencies. This preference may stem from their increased likelihood of encountering complications such as delivery hemorrhages, perineal tears, puerperal infections, and cervical lacerations.^40^ A possible explanation is that larger families, with at least four children, may have limited financial resources to afford institutional delivery.^37^

 This study’s primary strength lies in its comprehensive analysis of extensive data, producing detailed insights about Indonesia. However, as the study utilized secondary data, the scope of assessed variables was restricted to those available in the dataset. Consequently, the study did not include variables such as distance to healthcare facilities, cultural norms, quality of healthcare services, and levels of social support that may influence women’s decisions regarding institutional childbirth. Furthermore, due to limitations in the availability of disaggregated urban poor data at the provincial level within the national dataset, this study could not perform a more detailed regional analysis. Therefore, it is suggested that future research investigate this issue further.

**Highlights**

- Younger and divorced/widowed women were more vulnerable to non-institutional delivery.
- The poorest were more likely to use institutional delivery than those who were poorer.
- Insurance ownership significantly impacted the utilization of institutional delivery.
- Grand multiparous women had a higher likelihood of non-institutional delivery.

## Conclusion

 The study identified seven key barriers to institutional delivery:youngerage, low educational attainment, divorced or widowed marital status, unemployment, severe economic disadvantages, lack of insurance coverage, and grand multiparity. These findings suggest that improving access to institutional delivery services might require targeted interventions. Effective strategies may involve educational initiatives, financial assistance, and support systems to address the needs of younger, less educated, unemployed, and economically disadvantaged women. Additionally, enhanced healthcare policies and community-based support structures can help bridge existing service gaps.

 Developing instructional programs that highlight the benefits of institutional delivery is essential, particularly for women under 45, those with low education, and grand multiparas. Targeted support for divorced or widowed women, including counseling, peer groups, and community outreach, is also needed. Financial aid should be provided for unemployed and low-income women, including free or low-cost services, transport support, and subsidies. Moreover, expanding and improving access to health insurance through policy reforms is crucial to increasing coverage for uninsured women.

## Acknowledgments

 The authors would like to thank the Ministry of Health of the Republic of Indonesia for providing access to the data used in this study.

## Competing Interests

 The authors declare no conflict of interests regarding this manuscript.

## Ethical Approval

 This study was exempted from ethical review by the National Ethics Commission, as it involved secondary analysis of the 2023 Indonesian Health Survey data. Participation in this national survey was voluntary, and data were collected after obtaining informed consent from all respondents.

 The Dataset is not publicly available due to restrictions set by the legitimate data owner, the Indonesian Ministry of Health. However, eligible researchers can request access to the 2023 Indonesian Health Survey dataset via https://layanandata.kemkes.go.id/.

## Funding

 Not applicable.
